# New Insights into Nutrition and Gut–Liver Axis: A Focus on Non-Alcoholic Fatty Liver Disease

**DOI:** 10.3390/nu15234917

**Published:** 2023-11-25

**Authors:** Ildefonso Rodriguez-Ramiro

**Affiliations:** Department of Nutrition and Food Science, Faculty of Pharmacy, Complutense University of Madrid, 28040 Madrid, Spain; ildeforo@ucm.es

Non-alcoholic fatty liver disease (NAFLD) is one of the leading causes of chronic liver disease and represents a public health issue in Western industrialized countries. It encompasses a spectrum of diseases, including non-alcoholic fatty liver (NAFL) or steatosis, and non-alcoholic steatohepatitis (NASH). NAFL has a more benign prognosis, whereas NASH can progress to cirrhosis and hepatocellular carcinoma (HCC) [1]. Given that NAFLD is related to the obesity and diabetes epidemic, as well as other cardiometabolic risks, experts in the field have recently proposed changing the nomenclatures of NAFLD to metabolic dysfunction-associated steatotic liver disease (MASLD) and NASH to metabolic dysfunction-associated steatohepatitis (MASH) [2]. Since the planning of this Special Issue started before this recent proposal, we will keep the “traditional” NAFLD/NASH nomenclature, although the term “metabolic dysfunction” is of great interest.

The pathological progression of NAFLD is considered a “multiple hit theory” with a number of insults acting together to induce NAFLD progression. These hits include nutritional factors, insulin resistance, hormones secreted from the adipose tissue, oxidative stress damage, epigenetic factors, and endotoxins released by the gut microbiota [3]. In recent years, the impact of nutrition on the gut–microbiome–liver axis has emerged as one of the most important factors involved in the progression from simple steatosis to non-alcoholic steatohepatitis (NASH) or a more pathological liver phenotype. A chronic overload of fat and carbohydrates, and a low fiber intake can induce intestinal dysbiosis, an imbalance in gut microbial species abundance, which has been related to intestinal permeability. As a consequence, microbes or their metabolic products can reach the liver, promoting inflammation and the progression of NAFLD [4]. The pathophysiological role of intestinal permeability in hepatic chronic inflammation, one of the main NASH hallmarks, has been partly established. In brief, Kupffer cells (KC), a macrophage subset that act as the sentinels of the gut–liver interface, are activated in response to microbial products, initiating the recruitment of other immune cell populations through the release of chemokines [5]. However, the presence, regulation, and function of other immune populations, such as natural killer (NK) cells, dendritic cells, or B cells, in response to microbial products remain controversial [6]. Thus, more efforts are needed to fully elucidate the role of particular microbial gut populations and their products on the liver immune system during different NAFLD stages. For example, new insights introduce the possibility of identifying microbiome profiles as signatures of NAFLD progression in order to assess the severity of this pathology and the risk of its progression [7,8]. The use of different approaches, such as cellular, organoid, and mouse models, as well as clinical studies, are relevant to address future research on this topic.

Bioactive dietary compounds have been reported to prevent hepatic damage in NAFLD, counteracting some of the insults of the “multiple hit theory”. There is a lot of research focused on polyphenols [9], *n*-3 fatty acids [10], and polysaccharides [11], including dietary fibers [12], which have targeted a number of metabolic pathways involved in NAFLD progression. However, there are unresolved questions about the importance of bioactive compounds and their metabolites in gut–liver crosstalk. Since metabolic dysfunctions contribute to NAFLD progression, further research is needed (a) to fully describe new altered pathways in the gut–liver axis, and more importantly, (b) to find compounds that can target these metabolic abnormalities in NAFLD. Likewise, there is still an underexplored area of research about the impact of bioactive compounds on gut microbiota. The use of the new omics techniques will contribute to understanding the metabolic relevance of bioactive compounds in the modulation of gut microbiota as a therapeutic approach in NAFLD amelioration.

A healthy diet includes a varied list of foods which provides a combination of nutrients and/or compounds that may interact together. Thus, an interesting nutritional perspective concerns synergisms and the additive effects of bioactive dietary compounds. For example, we described the independent and additive effects of a combination of flavan-3-ols from cocoa polyphenols and *n*-3 fatty acids from fish oil, in NAFLD prevention. We observed that, in combination, these compounds ameliorated NAFLD via independent mechanistic effects. The combination of these compounds was able to mechanistically reduce the gene expression level of de novo lipogenesis, reduce triglycerides accumulation and hepatic steatosis, improve the insulin sensitivity, and change the bile acids metabolism profile [13]. Thus, this Special Issue is also a call for research aiming to explore the additive and/or combinatory effects of bioactive compounds from diets, targeting altered metabolic pathways.

Last but not least, the pathogenesis of NAFLD progression in patients with inflammatory bowel diseases (IBD) is not yet understood. Nevertheless, more than one-third of patients with inflammatory bowel diseases (IBD) are affected by extraintestinal manifestations, which include NAFLD [14]. A recent clinical trial has reported that NAFLD and advanced fibrosis were significantly more prevalent in the IBD population than they were in the general population. This study suggests IBD as an independent factor explaining the severity of NAFLD progression [15]. However, previous research by Ritaccio et al. observed that the risk of liver fibrosis progression in IBD patients with NAFLD is low [16]. Thus, the debate about whether IBD is an independent risk factor of NAFLD progression is still open, and it deserves more research focused on mechanistic studies to clarify this controversy.

In all, this Special Issue is a call for state-of-the-art research aiming to unravel novel insights of nutrition and metabolism in NAFLD prevention/treatment in the context of the gut–liver axis.
